# Materials Nanoarchitectonics at Dynamic Interfaces: Structure Formation and Functional Manipulation

**DOI:** 10.3390/ma17010271

**Published:** 2024-01-04

**Authors:** Katsuhiko Ariga

**Affiliations:** 1 Research Center for Materials Nanoarchitectonics (MANA), National Institute for Materials Science (NIMS), 1-1 Namiki, Tsukuba 305-0044, Ibaraki, Japan; ariga.katsuhiko@nims.go.jp; 2 Graduate School of Frontier Sciences, The University of Tokyo, 5-1-5 Kashiwanoha, Kashiwa 277-8561, Chiba, Japan

**Keywords:** assembly, dynamic interface, fullerene, Langmuir–Blodgett film, liquid, nanoarchitectonics, thin film

## Abstract

The next step in nanotechnology is to establish a methodology to assemble new functional materials based on the knowledge of nanotechnology. This task is undertaken by nanoarchitectonics. In nanoarchitectonics, we architect functional material systems from nanounits such as atoms, molecules, and nanomaterials. In terms of the hierarchy of the structure and the harmonization of the function, the material created by nanoarchitectonics has similar characteristics to the organization of the functional structure in biosystems. Looking at actual biofunctional systems, dynamic properties and interfacial environments are key. In other words, nanoarchitectonics at dynamic interfaces is important for the production of bio-like highly functional materials systems. In this review paper, nanoarchitectonics at dynamic interfaces will be discussed, looking at recent typical examples. In particular, the basic topics of “molecular manipulation, arrangement, and assembly” and “material production” will be discussed in the first two sections. Then, in the following section, “fullerene assembly: from zero-dimensional unit to advanced materials”, we will discuss how various functional structures can be created from the very basic nanounit, the fullerene. The above examples demonstrate the versatile possibilities of architectonics at dynamic interfaces. In the last section, these tendencies will be summarized, and future directions will be discussed.

## 1. Introduction

In considering the development of functional materials, it is significant to learn how a biological system creates a functional system. This is because in a biofunctional system, multiple components are rationally arranged, and they selectively perform their functions with a very high efficiency [1,2,3]. Each function is very dynamic, and several functions work in harmony. Such a high level of functional expression remains difficult to achieve in artificial materials systems. Many functions in biological systems are achieved by the accumulation of functional molecules at interfaces such as cell membranes [4,5,6,7]. Intermolecular interactions work together at these interfaces, and information such as electrons and energy flow in specific directions. Biological systems have established these superior mechanisms over billions of years of evolution. Most of these events are conducted at dynamic interfaces. The construction of material systems at dynamic interfaces will be the key for humans to imitate them in a short time and to develop advanced functional systems.

On the other hand, humanity has developed functional materials in the course of various scientific developments. The history of the development of functional materials corresponds to the history of human progress. Even today, the development of various functional materials is still necessary to meet the demands of energy [8,9,10,11,12,13,14,15,16,17], the environment [18,19,20,21,22,23,24,25,26,27], and biomedical requirements [28,29,30,31,32,33,34,35,36,37]. Modern science and technology must develop materials for various functions, such as for energy production [38,39,40,41,42,43,44,45], energy storage [46,47,48,49,50,51,52], separation [53,54,55,56,57,58,59,60], sensing [61,62,63,64,65,66,67,68], and drug delivery [69,70,71,72,73,74,75,76]. In the 20th century, academic areas for the creation of various materials have been established, and it has become possible to create materials on demand that do not exist in nature. This is supported mainly by materials-related chemistry. Humans have developed various types of chemistry since the 20th century, which has led to modern scientific research. Research on the development of functional systems through organic chemistry [77,78,79,80,81,82,83,84,85,86], inorganic chemistry [87,88,89,90,91,92,93,94,95,96], coordination chemistry [97,98,99,100,101,102,103,104,105,106], supramolecular chemistry [107,108,109,110,111,112,113,114,115,116], polymer chemistry [117,118,119,120,121,122,123,124,125,126], interface chemistry [127,128,129,130,131,132,133,134,135,136], various material chemistry [137,138,139,140,141,142], and bio-related chemistry [143,144,145,146,147,148,149,150,151,152] has continued unceasingly.

The science of functional material development and structural control, such as the creation of hybrids and composites [153,154,155,156,157,158], has raised awareness of the importance of not only the material itself, but also its internal nanostructure [159,160,161]. The turning point was the creation of the concept of nanotechnology. Nanotechnology has been advancing science and technology with the symbolic size of the nanoscale. It is continuously active today. We observe and manipulate objects at the atomic, molecular, and nanoscale levels [162,163,164,165,166,167]. Furthermore, we can analyze and understand phenomena in those nanoregions [168,169,170,171]. Such technologies have been applied to analytical chemistry [172,173,174,175,176] and physical chemistry [177,178,179,180,181], and the impact of the structure of a material on its function has been studied.

**Figure 1:**
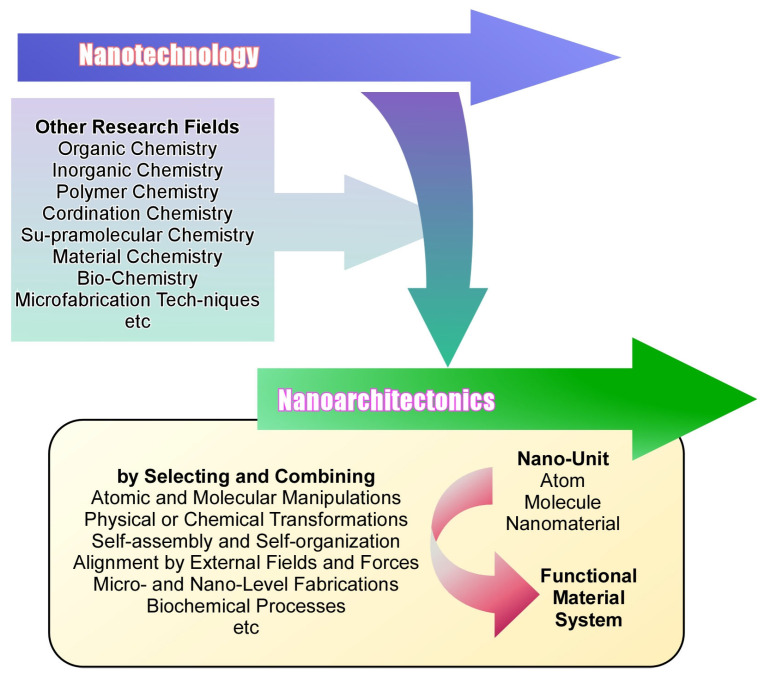
Outline of nanoarchitectonics, which combines nanotechnology with organic chemistry, inorganic chemistry, polymer chemistry, coordination chemistry, supramolecular chemistry, material chemistry, biochemistry, and microfabrication techniques.

The next step in nanotechnology is to establish a methodology to assemble new functional materials based on the knowledge of nanotechnology. This task is taken by nanoarchitectonics [182,183,184,185]. Just as Richard Feynman founded nanotechnology in the 20th century [186,187], nanoarchitectonics was proposed by Masakazu Aono at the beginning of the 21st century [188,189]. Nanoarchitectonics can be considered as a post-nanotechnology concept [190]. This concept is not something completely new, but rather represents the integration of established fields: it combines nanotechnology with organic chemistry, inorganic chemistry, polymer chemistry, coordination chemistry, supramolecular chemistry, material chemistry, biochemistry, and microfabrication techniques (Figure 1) [191].

In nanoarchitectonics, we architect functional material systems from nanounits such as atoms, molecules, and nanomaterials. It architects functional materials through the selection and combination of atomic and molecular manipulations, physical or chemical transformations, self-assembly and self-organization, alignment by external fields and forces, microlevel and nanolevel fabrications, and biochemical processes [192]. With this method of combining several processes, hierarchical structures are more easily obtained [193] than in the process of self-assembly by simple equilibrium [194,195,196]. High functionality in biological systems is due to the flow of molecules, ions, electrons, and energy in hierarchical and asymmetric structures. Nanoarchitectonics is predisposed to construct such advanced concepts in biological systems [197]. In addition, nanolevel phenomena are prone to contain uncertainties. They may not be uniquely determined by thermal fluctuations, stochastic distributions, quantum effects, etc. [198]. The effects tend to be harmonized rather than summarized [199]. Nanoarchitectonics, which is founded on nanophenomena, has the characteristic of harmonizing effects. It also has similar characteristics to biofunctional systems, where the effects are dynamically harmonized in thermal fluctuations. In terms of the hierarchy of the structure and the harmonization of the function, material creation by nanoarchitectonics has similar characteristics to the organization of the functional structure in biosystems [200].

In biofunctional systems, a wide variety of functional molecules are organized. In order to mimic such systems, it is essential to have a material-creating capability for every object. In principle, nanoarchitectonics can be applied to any material without any limitations. All matter is composed of atoms and molecules. Nanoarchitectonics, which builds materials from molecules and atoms, is a methodology that can be applied to the creation of all materials. It can be a Method for Everything in material chemistry [201,202], analogous to the Theory of Everything in physics [203]. In fact, nanoarchitectonics has a wide range of applications. The research papers that claim to be nanoarchitectonics are as follows. The application of nanoarchitectonics can be seen in basic fields, such as the synthesis of functional materials [204,205,206,207,208,209,210,211,212,213,214,215,216,217,218,219], the control of specific structures [220,221,222,223,224,225,226,227,228,229,230], the creation of structures [231,232,233,234,235,236,237,238,239,240], basic physics [241,242,243,244,245,246,247,248,249,250,251,252], relatively basic biochemistry [253,254,255,256,257,258,259,260], and the development of simple functional materials for biological systems [261,262,263,264,265,266,267,268,269,270,271,272], as well as application-oriented fields such as catalyst development [273,274,275,276,277,278,279,280,281,282], sensors [283,284,285,286,287,288,289,290,291], devices [292,293,294,295,296,297,298,299], energy production [300,301,302,303,304,305,306,307], energy storage [308,309,310,311,312,313,314,315,316,317], the environment [318,319,320,321,322,323,324,325,326,327], biomedical [328,329,330,331,332,333,334,335,336,337,338,339,340,341], etc. This can be regarded as a list of some of the most important applications of the current technology.

The characteristics of nanoarchitectonics, such as hierarchical structure creation, function harmonization, and deployment in a variety of materials, are suited to the architecture of bio-like functional structures. Looking at actual biofunctional systems, as mentioned above, the dynamic properties and interfacial environments are important keys. In other words, nanoarchitectonics at dynamic interfaces is important. In particular, thin film fabrication at the interface will be an important methodology for this target [342,343,344]. One method for forming dynamic structures at interfaces that do not move, such as individuals, is the vacuum deposition method [345,346,347]. Thin film nanoarchitectonics has also been used as a thin film creation material, such as with DNA-related materials that exhibit dynamic properties [348]. Layer-by-layer (LbL) assembly is a well-known methodology for the nanoarchitectonics of thin films on solid substrates or colloidal particles in a wet process [349,350,351,352,353]. In this technique, a very wide range of materials are sequentially stacked in the desired manner with a simple operation. The interfaces where adsorption occurs are mostly solid substrates or solid colloids, which do not move dynamically. However, the thin films that are stacked are mostly amorphous and have dynamic motion and function [354,355,356]. A typical example of a thin film preparation method that uses a dynamic interface with free motion is the Langmuir–Blodgett (LB) method, which uses a gas–liquid interface [357,358,359]. Thin films deployed on a liquid interface (mainly a water surface) are freely compressed, aligned, controlled, and organized. The thin films can be transferred to a solid substrate as a multilayer structure [360,361,362]. In recent years, the vortex LB method, which promotes orientation by introducing a vortex flow at the air–water interface, has also been devised [363]. Ultra-high temperature LB methods have also been developed that use conditions of 100 °C [364] or 200 °C [365], rather than the usual room temperature conditions. Organizing methods using not only the air–water interface but also the liquid–liquid interface have also been reported [366,367]. These methods can be called nanoarchitectonics methods using dynamic interfaces.

Nanoarchitectonics is the concept of architecting materials from nanounits. The ultimate model for reference is the highly organized functional structure of biological systems. The big challenge of nanoarchitectonics is related to the establishment of a unified concept that produces bio-like functional materials systems from essential atoms, molecules, and nanounits, rather than accomplishments in particular fields. The key to this is the interfacial environment and its dynamic nature. In this review paper, nanoarchitectonics at dynamic interfaces will be discussed, looking at typical recent examples. In particular, the more basic topics of “molecular manipulation, arrangement, and assembly” and “material production” will be discussed in the first two sections. Then, in the following section, “fullerene assembly: from zero-dimensional unit to advanced materials”, we will discuss the creation of various functional structures from the very basic nanounit, the fullerene. These examples will demonstrate the versatile possibilities of architectonics at dynamic interfaces. In the last section, these trends will be summarized, and future directions will be discussed.

## 2. Molecular-Level Dynamic Process: Manipulation, Arrangement, and Assembly

Compared to crystals and solid surfaces, the degree of freedom of molecular motion is much higher in liquid systems. In a solution system spread over three dimensions, the degree of freedom of molecular motion is too large to control. Unlike three-dimensional systems, liquid interfaces such as gas–liquid and liquid–liquid are used as externally controllable fields that preserve the degree of freedom of molecular motion by the liquid. In particular, the Langmuir–Blodgett method has been used to control molecules at the gas–water interface in various ways.

Negi et al. synthesized some variously designed chiral molecules and analyzed their behavior on the water surface to control their molecular orientation and to verify the molecular structures that are suitable for supramolecular structures and nanostructures [368]. The compound consists of a pyrene ring introduced into the asymmetric carbon portion of a secondary alcohol, which is the hydrophilic portion of an amphiphilic molecule with a pyrene sulfonic acid group. As concrete molecules, 1-stearylpyrene-6-sulfonate and 1-stearylpyrene-8-sulfonate were synthesized, and their monolayer behavior was analyzed. In addition to the conventional method of π-A isotherms, in situ surface fluorescence spectroscopy, Brewster angle microscopy, and atomic force microscopy were used to study the assembling behavior. In particular, the structures of the racemic and optically active monolayers were analyzed. The monolayers that formed at the air–water interface of the racemic compounds resembled a racemic solid solution. Furthermore, the optically active compound formed a monolayer with a well-controlled structure. This is due to the cooperative action of the face-to-face association of the pyrene rings and chiral steric factors. The combination of π-π stacking and chirality allows for some control of the monolayer structure. In order to control the structure of the molecular assemblies by dynamic movements at the interface, it is important to use chirality more effectively. For this purpose, further improvement of the molecular design is needed. To control the molecular assemblies more precisely, it is also important to combine chirality with other intermolecular interactions, a combination that is useful for the design of supramolecular structures and nanostructures.

**Figure 2:**
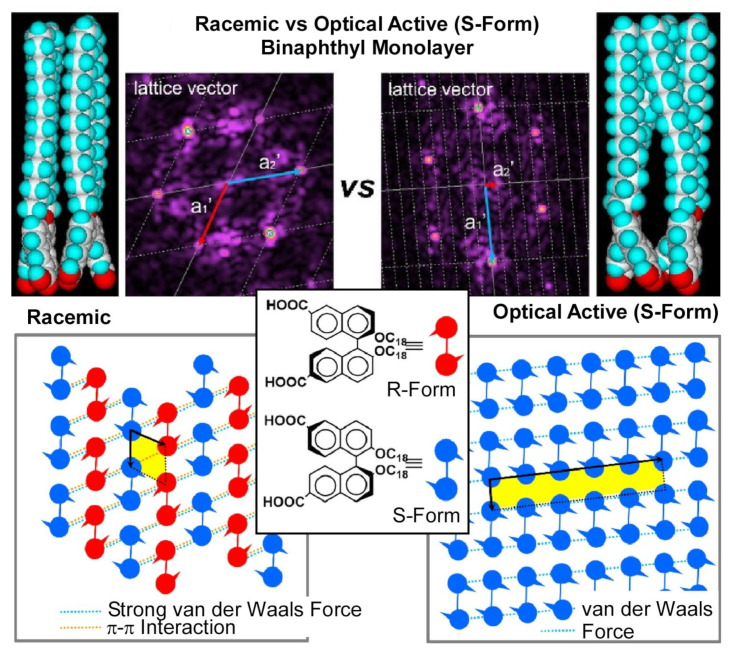
Two-dimensional nanoarchitectonics of binaphthyl molecules with an axial chirality in its racemic form (**left**) and its optically active S-isomer form (**right**). Reprinted with permission from [369]. Copyright 2023, Chemical Society of Japan.

Negi et al. also investigated the pressure dependence of the aggregation nanoarchitectonics of binaphthyl-type amphiphiles at dynamic interfaces [369]. In this study, 2, 2′-bis(octadecyloxy)-1, 1′-binaphthyl-6, 6′-dicarboxylic acid was designed as a monolayer-forming molecule. This molecule has an axial chirality derived from the binaphthyl moiety with two COOH groups. The monolayer behavior of the racemic and optically active S forms was investigated. Brewster angle microscopy, atomic force microscopy, and surface pressure–area isotherms were used to analyze the monolayer structure. The racemic form creates a solid film, while the S-isomer forms a liquid film. It was also found that the real lattices of these monolayers differ significantly (Figure 2). The differences in the real lattice are due to steric regularity resulting from axial chirality, which causes differences in the mode of intermolecular interactions. In the racemic case, the solid membrane is formed by a cooperative network of π-π interactions between the naphthyl rings and of strong van der Waals interactions. This model is consistent with the actual lattice obtained from atomic force microscopic images. In the S-isomer, the intermolecular distance is greater than in the racemic form, so strong intermolecular forces are less likely to act. Relatively weak interactions are expected to result in a one-dimensional arrangement of long alkyl chains and naphthyl rings. At a low surface pressure, the van der Waals interactions between the monolayers are small and the intermolecular forces are relatively weak. However, upon compression, a phase transition from liquid-expanded film to liquid-condensed film was observed at the π-A isotherm. It is considered that the two-dimensional physical compression at the dynamic interface reduced the dihedral angle of the binaphthyl ring in the one-dimensional columnar structure, narrowing the intermolecular distance and thus strengthening the intermolecular interactions, which induced the phase transition.

**Figure 3:**
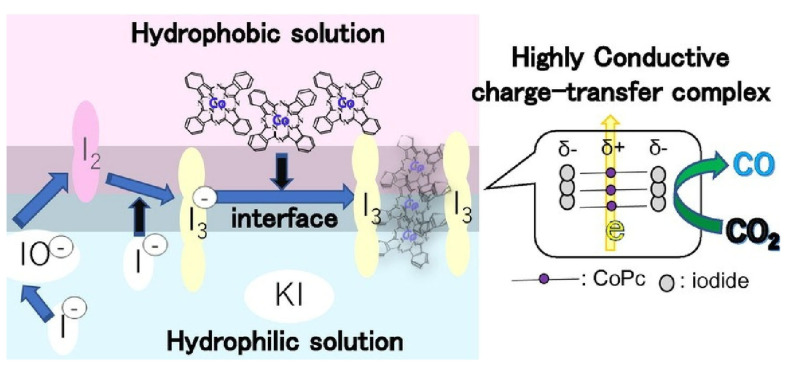
The nanoarchitectonics of the highly conductive charge transfer complex of (phthalocyaninato)cobalt iodide through a cobalt phthalocyanine crystal phase transition method by simply mixing a KI solution containing CF_3_COOH and cobalt phthalocyanine with a CH_2_Cl_2_ solution at the interface. Reprinted with permission from [370]. Copyright 2023, Chemical Society of Japan.

Takase et al. focused on a highly conductive charge transfer complex of (phthalocyaninato)cobalt iodide [370]. They investigated the effect of the enhanced conductivity of cobalt phthalocyanine crystals on catalytic activity. This is also beneficial for the industrial application of cobalt phthalocyanine as a catalyst for CO_2_ reduction. They applied the cobalt phthalocyanine crystal phase transition method to develop a useful method for synthesizing (phthalocyaninato)cobalt iodide by simply mixing a KI solution containing CF_3_COOH and cobalt phthalocyanine with a CH_2_Cl_2_ solution at the interface (Figure 3). UV-vis absorption spectra showed that under acidic conditions, the iodide ion (I^−^) changes to triiodide ion (I_3_^−^) at the interface between the aqueous potassium iodide solution and the organic solvent. In other words, when the aqueous KI solution was mixed with an organic acid solution containing cobalt phthalocyanine, the I^−^ changed to I_3_^−^, forming a highly conductive charge transfer complex of (phthalocyaninato)cobalt iodide upon cobalt phthalocyanine with the I_3_^−^ stacked separately. The catalytic properties of (phthalocyaninato)cobalt iodide were investigated with polarization measurements and electrochemical impedance spectroscopy using a gas diffusion carbon electrode. The (phthalocyaninato)cobalt iodide nanoarchitectonized at the dynamic interface and showed high catalytic activity for CO_2_ reduction and high CO formation selectivity. It is expected that such attempts can be applied to electrochemical devices.

**Figure 4:**
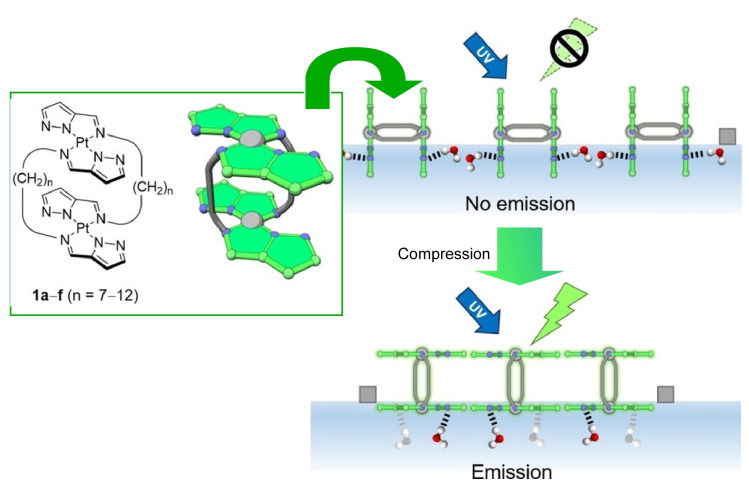
A new principle, submarine luminescence, as a luminescence control, involving molecular manipulation of double-paddle platinum complexes through compression at the air–water interface. Reprinted with permission from [375]. Copyright 2020, Wiley-VCH.

Dynamic interfaces can also be a field for molecular manipulation [371,372]. For example, the coupling of macroscopic mechanical manipulations and molecular functions, which are phenomena that differ greatly in size, is possible in a field with reduced dimensions, such as in a field like a dynamic interface [373,374]. In an asymmetric interfacial two-dimensional system such as a molecular thin film, the film has a macroscopic dimension in the in-plane direction and a molecular dimension in the thickness direction. Mechanical macroscopic motions in the in-plane direction of the monolayer may be reflected in the molecular function. By compressing the monolayer by tens of centimeters, nanometer-sized molecular machines and structures can be easily controlled. For example, Adachi et al. have developed a new principle called “submarine luminescence” as a luminescence control, involving the molecular manipulation of double-paddle platinum complexes at the air–water interface (Figure 4) [375]. When a monolayer of the double-paddle Pt complexes is formed at the air–water interface and the water surface is compressed from both ends, the luminescence intensity increases rapidly from a certain compression state. The double-paddle platinum complexes are used to form square planar complexes. These double-paddle platinum complexes exhibit unique molecular activities such as flapping and the relative rotation of the double plane, and the luminescent sites of the double-paddle platinum complexes float from the aqueous phase, a high dielectric medium, to the gas phase, a low dielectric medium. The luminescence intensity increases as the luminescent sites levitate from the aqueous phase. Spectroscopic measurements and DFT calculations confirm that the molecular arrangement pattern changes simultaneously with the luminescence switching. The luminescence ability of the monolayer is determined by whether the luminescent sites are present in water or air. At a low surface pressure, the H-shaped complex of the double-paddle platinum complex sinks in water and has a weak emission due to hydrogen bonding. The double-paddle platinum complex is pushed to the surface by mechanical compression, so that the two planes behave independently at the air–water interface. One face in the aqueous phase is suppressed, while the other face in the gas phase is freed from the excitation energy dispersion due to the molecular contact. This is an example of using the difference between two adjacent environments, the aqueous and gas phases, to control physical properties. This is a “submarine luminescence” mechanism, in which the optical properties are controlled in a submarine-like manner through molecular manipulation in a nanometer-sized asymmetric interfacial environment. This opens up new possibilities for molecular manipulation using the dynamic behavior of the air–water interface.

**Figure 5:**
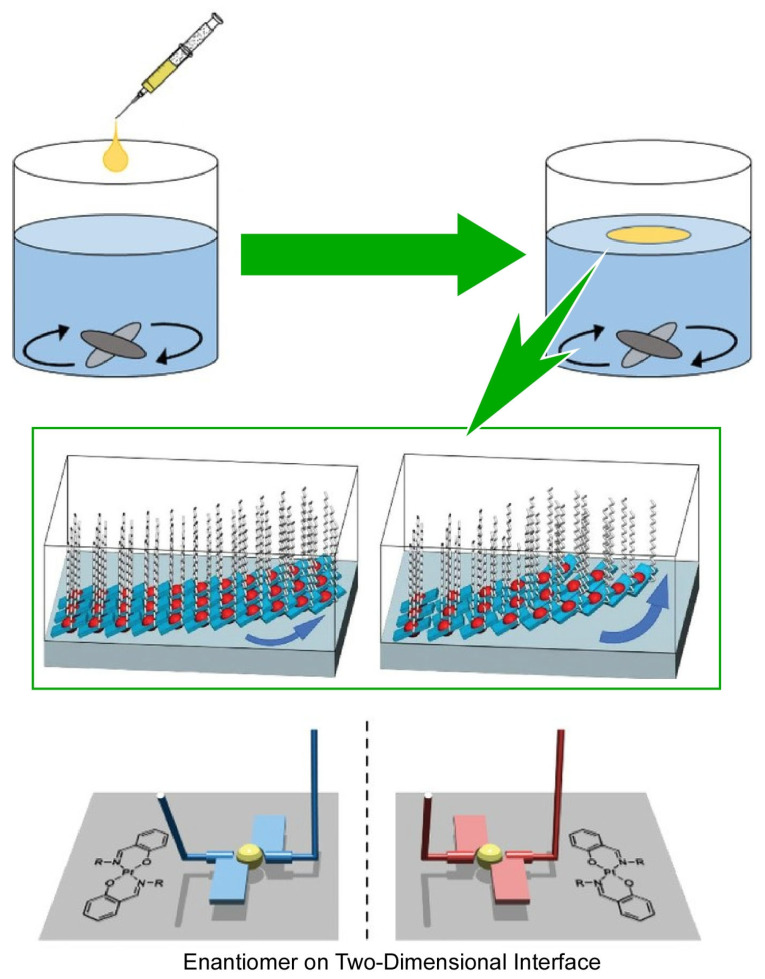
A flexible control of the intensity and signature of circularly polarized emissions from molecular aggregates of an achiral trans-bis(salicylaldiminato)platinum(II) complex upon precisely controlled clockwise and counterclockwise vortex motions at the air–water interface. Reprinted with permission from [376]. Copyright 2022, Wiley-VCH.

Maeda et al. reported a method that allows flexible control of the intensity and signature of circularly polarized emissions from molecular aggregates at a dynamic interface with rotational motion (Figure 5) [376]. In this study, an achiral trans-bis(salicylaldiminato)platinum(II) complex is deployed at the air–water interface. The circularly polarized luminescence of the aggregates was precisely controlled by creating clockwise and counterclockwise vortexes at the air–water interface and by specifically aggregating them at various motion velocities. The two-dimensional aggregates formed at the air–water interface under the vortex flow are quite different from the three-dimensional molecular arrangement of crystals formed from organic solutions. The helical torsional forces of the vortex flow result in the supramolecular chirality of the two-dimensional domains caused by the one-way torsion in the coordination plane stacking. The sense of torsion is determined by the direction of the vortex rotation, and the degree of torsion is controlled by the vortex velocity. The complete and infinite stacking of trans-bis(salicylaldiminato)platinum(II) coordination planes induces dispersion of the optical energy of the excited state. The platinum coordination planes remain in mutual contact with each other in a continuous, shallow stacking interaction during the chiral transition via helical twisting at the organic–water interface. The enhancement of luminescence by eddy current control is attributed to the change in the stacking status of the coordination planes depending on the vortex velocity. This method of nanoarchitectonics at dynamic interfaces is a milestone example of precisely controlled, circularly polarized emission from molecular aggregates.

**Figure 6:**
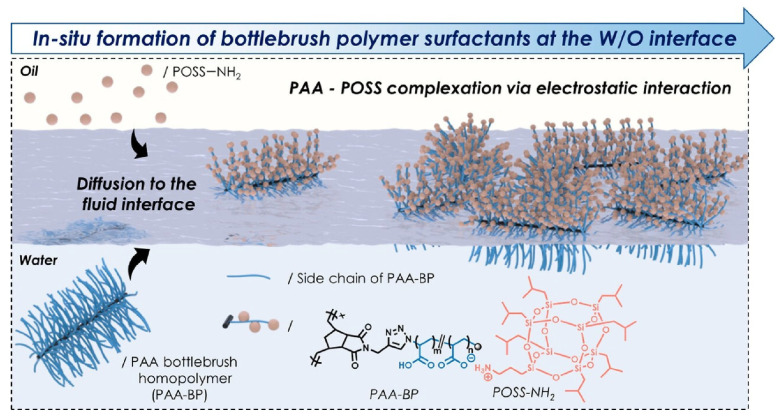
Aggregation behavior of bottlebrush polymers at dynamic interfaces, as formed by electrostatic interaction of carboxylate (poly(acrylic acid)) and ammonium (aminopropylisobutyl polyhedral oligomeric silsesquioxane). Reprinted with permission from [377]. Copyright 2023, American Chemical Society.

Emrick, Russell, and co-workers examined the aggregation behavior of bottlebrush polymers at dynamic interfaces (Figure 6) [377]. The aim of this study was to elucidate the fundamental relationships between the nanostructural and macroscopic properties of various bottle brush polymers. The results answer the question of how the properties of all fluid-printed structures can be controlled by molecular design and their properties. Bottle brush polymer surfactants are formed by the interfacial interaction between a bottle brush polymer with poly(acrylic acid) side chains dissolved in the aqueous phase and an amine functionalized ligand dissolved in the oil phase. The bottlebrush polymer surfactants were formed at the water–oil interface through the electrostatic interaction of carboxylate (poly(acrylic acid)) and ammonium (aminopropylisobutyl polyhedral oligomeric silsesquioxane). The bottlebrush polymer surfactant strongly associates and binds to the liquid–liquid interface. The initial aggregation rate, interface filling efficiency, and stress relaxation are specified by the ratio of the degree of polymerization of the backbone to that of the side chains. Bending stiffness as a mechanical property and stress relaxation behavior during compression of the aggregates were also studied. The ratio of the degree of polymerization of the polymerization side chains of the backbone changed the effective area projected on the fluid interface of the polymer from spherical to cylindrical. The results provide general design guidelines for the printing of structured liquids with bottle brush polymers. In addition, the high density of functional groups at the printed liquid interface of the bottlebrush polymer interfacial activity allows for further hierarchical nanoarchitectonics, such as incorporating metal cations and active enzymes. These structures could also be applied to applications such as two-phase reactors where interfacial chemistry can be controlled.

A dynamic interface provides a field in which the degree of freedom of motion is guaranteed to some extent and does not diverge significantly. In such a field, it is possible to manipulate molecules and create unique molecular assemblies by external forces. For example, external forces can manipulate the molecular position between the two phases of air and water to control the luminescence behavior. The direction and intensity of the vortex flow at the interface can tune the circularly polarized luminescence of the assembly mode of the functional complex molecules. It is expected that the successful combination of appropriate molecular design and dynamic interfacial nanoarchitectonics will enable a wide variety of molecular manipulation and assembly control.

## 3. Material Production: Dynamic Materials-Level Formation Process at Interfaces

At dynamic interfaces, it is possible to manipulate molecules and control their aggregate morphologies. When those molecules react or combine, they become materials. This also happens with inorganic materials and their hybrids. Dynamic interfaces provide an appropriate site for material production, not just limited to molecular assembly.

**Figure 7:**
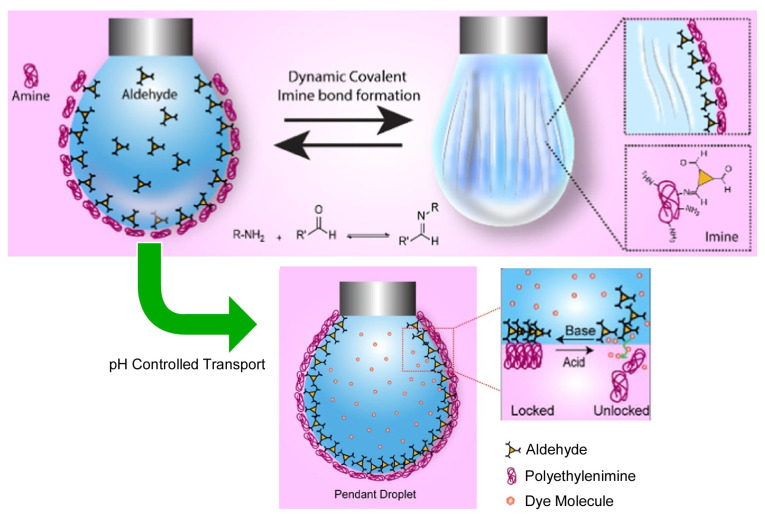
Dynamic covalent bonding controlled through modulating the droplet shape at the dynamic interface, where anisotropic compartmentalization of the liquid–liquid interface thus occurs, and the pH dependence of the Schiff base reaction leads to reversible toggling from jamming to unjamming in the interfacial assembly. Reprinted with permission from [381]. Copyright 2022, American Chemical Society.

Dynamic interfaces, such as the liquid–liquid interface, provide a very good venue for creating biomimetic structures such as artificial cell membranes [378,379,380]. One of the candidates for the creation of biomimetic structures is cell membrane models. In such models, permeability controls through cell-mimic membranes are attractive research targets. Patra and co-workers used dynamic imine chemistry at the oil–water interface to control mass permeability (Figure 7) [381]. The effect of dynamic covalent bonding was controlled through modulation of the droplet shape at the dynamic interface. Imine bond formation between water-soluble polyethyleneimine and oil-soluble aromatic aldehydes significantly reduces the interfacial tension and greatly stabilizes the oil–water interface. Furthermore, the interfacial tension can be tuned through varying the degree of aldehyde (mono-, di-, and tri-) functionality in the imine bond formation. When a compressive force is applied to the droplet, the imine-mediated aggregation is successfully packed. Anisotropic compartmentalization of the liquid–liquid interface thus occurs. The pH dependence of the Schiff base reaction is skillfully exploited to shift the equilibrium, leading to reversible toggling from jamming to unjamming in the interfacial assembly. The stimuli-responsive behavior of the dynamic imine assembly was demonstrated in a pH-triggered control of content release through the interfacial membrane. At a pH of 10, maximal cross-linking through the imine bonds occurred at the interface. As a result, the diffusion of the dye molecules through this interfacial membrane was greatly inhibited (the locked state). On the other hand, at a pH of 2, the opposite condition occurred (the unlocked state), where the interfacial cross-linking was negligible and the dye release was maximal. These results are expected to contribute significantly to the field of dynamic covalent assembly at the liquid–liquid interface, droplet structuring, and various types of stimuli-responsive membranes.

**Figure 8:**
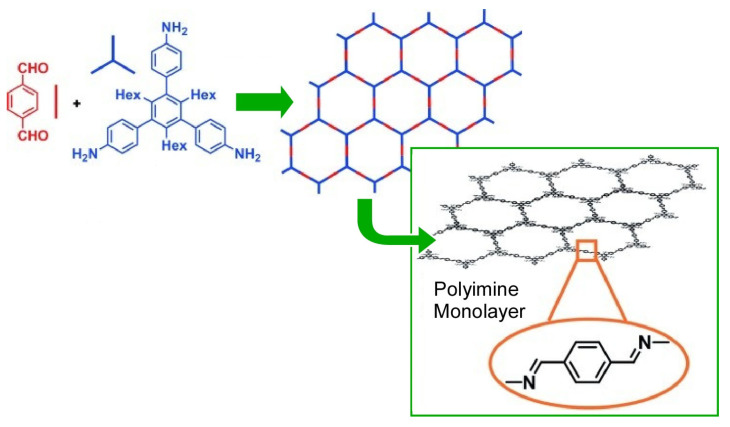
Covalent two-dimensional polymer under ambient temperature conditions at the air–water interface with the thickness of the sheet corresponding to that for its monolayer. Reprinted with permission from [382]. Copyright 2016, Wiley-VCH.

Dynamic imine chemistry, which deals with the Schiff base formation, is one promising approach for the synthesis of well-defined molecular structures. In imine chemistry, the dynamic imine bond formation properties allow for a self-healing mechanism. Thus, molecular structures can be formed with thermodynamic control. Widely applied to small molecule and polymer synthesis, it is useful for the synthesis of two-dimensional polymers at interfaces. Zhang and co-workers synthesized a covalent monolayer of two-dimensional polymer under ambient temperature conditions at the air–water interface (Figure 8) [382]. The prepared two-dimensional polymer sheets, which were transferred from a Langmuir trough via a horizontal Schaeffer-type transfer. The thickness of the sheet (0.7 nm), determined by atomic force microscopy (AFM), corresponded to that for its monolayer. A freestanding monolayer sheet was obtained, with lateral dimensions in the range of tens of micrometers. Within the resolution range, the presence of imine bonds and the absence of terminal groups were indicated through the Raman spectra. Smooth, coherent, large, freestanding polyimine monolayers were nanoarchitectonized. Such nanoarchitectonics of conjugated two-dimensional polymers at dynamic interfaces will contribute to providing materials for electronic and optoelectronic applications.

**Figure 9:**
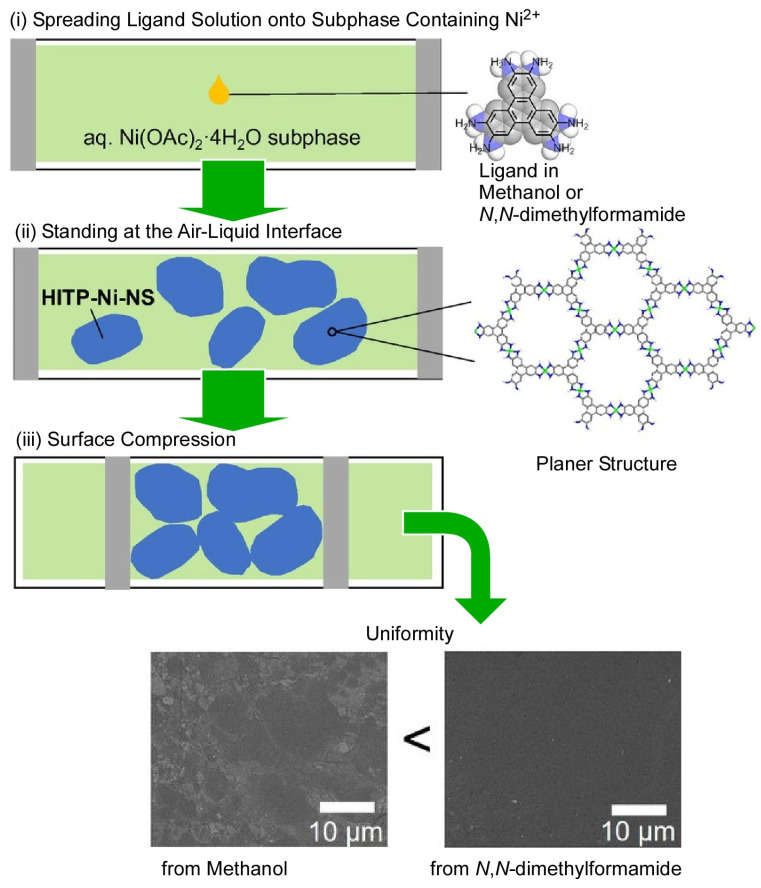
Effect of the type of solvent (methanol or *N*,*N*-dimethylformamide) in the ligand diffusion solution to control the formation of MOF nanosheets synthesized through spreading the ligand 2,3,6,7,10,11-hexaiminotriphenylene on an aqueous solution containing Ni^2+^. Reprinted with permission from [388]. Copyright 2023, Chemical Society of Japan.

As described above, material synthesis at the dynamic interface, the air–liquid interface, is a promising method for obtaining nanosheets because the components grow spontaneously in a two-dimensional direction. Various types of nanosheets have been synthesized at dynamic interfaces from various functional molecules. Metal–organic framework (MOF) and covalent organic framework (COF) nanosheets are nice examples [383,384,385,386,387]. MOF nanosheets have attracted significant attention as components of electronic devices such as electrocatalysts and chemical resistivity sensors. However, achieving the desired properties with reasonable control over size and shape is not always easy. Makiura and co-workers investigated the effect of the type of solvent in the ligand diffusion solution to control the formation of the MOF nanosheets (Figure 9) [388]. In this MOF nanosheet synthesis, the ligand 2,3,6,7,10,11-hexaiminotriphenylene was spread on an aqueous solution containing Ni^2+^. The influence of the solvents used, methanol and *N*,*N*-dimethylformamide, was investigated. The usual role of solvents in the Langmuir film formation is to disperse molecules at the air–liquid interface without causing aggregation. In addition to this, the dynamic synthesis of the MOF nanosheets at the air–water interface also involves effects on coordination bonds and p-p interactions. Different expansion solvents alter the uniformity, morphology, lateral domain size, and chemical composition of the nanosheets. The macroscopic morphological uniformity of the MOF nanosheets is higher when *N*,*N*-dimethylformamide is used as the expansion solvent than when methanol is used as the solvent. The evaporation of the highly volatile methanol results in a variety of interfacial states, including surface ripping and solvent subphase miscibility. This results in variations in morphology on the micrometer scale. Conversely, *N*,*N*-dimethylformamide is less volatile and easily binds to the amino groups of the ligand. This facilitates the formation of coordination bonds and the growth of nanosheets. These insights into MOF nanosheet synthesis at dynamic interfaces provide the fundamental knowledge needed to further optimize interfacial synthesis methods. Full control of the nanoarchitectonics of MOF nanosheets will enable the prediction of chemical and physical properties closely related to the properties of the nanosheets. This will accelerate their use in diverse nanodevices.

**Figure 10:**
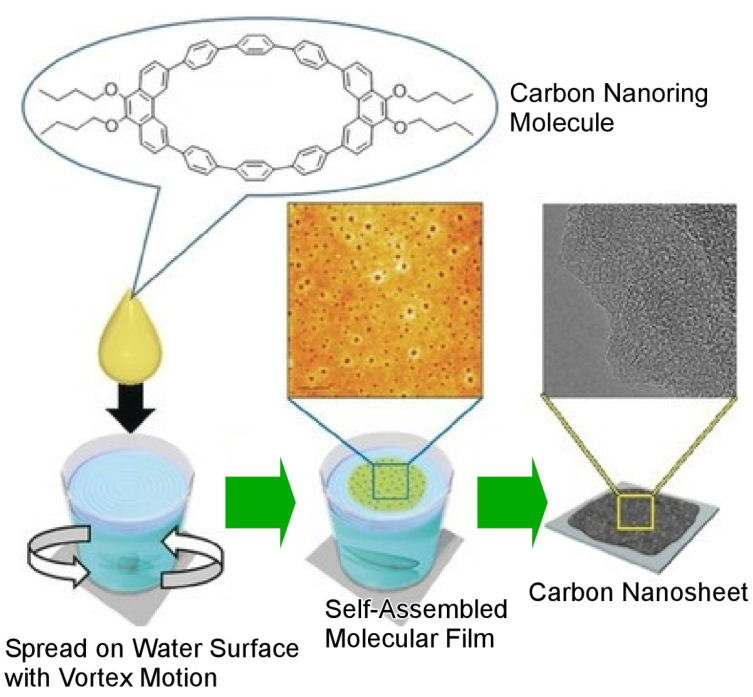
Fabrication of mesoporous thin films with both uniformity and many tens of nanometer pores through a simple method in which a vortex flow is created in a beaker filled with water and carbon nanorings that float on the water surface and are then transferred to a substrate. The resulting thin films were carbonized under an inert gas atmosphere to synthesize carbon nanosheets. Reprinted with permission from [392]. Copyright 2018, Wiley-VCH.

The dynamic interface is an ideal place to synthesize two-dimensional materials. Carbon materials such as carbon nanosheets, represented by graphene, are attracting attention as novel electronic, optical, and catalytic materials [389,390,391]. In addition to conventional methods, the bottom–up method of carbon nanosheet synthesis, in which molecules are assembled into carbon through self-assembly, is attracting attention. The advantage of this bottom–up method is that the structure formed by the molecules can be precisely controlled at the nanolevel. However, the bottom–up method requires knowledge of advanced supramolecular chemistry. Therefore, a method to obtain nanosheets using a simple technique that anyone can do is being sought. Mori et al. reported the fabrication of mesoporous thin films with both uniformity and many tens of nanometer pores through a simple method in which a vortex flow is created in a beaker filled with water and carbon nanorings, which are ring-shaped carbon molecules that float on the surface of the water and are then transferred to a substrate (Figure 10) [392]. A method that can create a vortex flow at the water surface and orientate and accumulate materials with that flow is called the vortex Langmuir–Blodgett method (vortex LB method). This is a nanoarchitectonics method that utilizes dynamic material movement at the interface. The resulting thin films were carbonized under an inert gas atmosphere to synthesize carbon nanosheets. Interestingly, this molecular thin film composed of carbon nanorings had numerous pores (mesoporous) of several tens of nanometers. Even after carbonization through calcination, the carbon nanosheets retained this mesoporous structure. Before calcination, the nanosheet is an insulator that does not conduct electricity. However, after calcination, the carbon nanosheets have been transformed into conductors. In other words, the carbon is bonded together through calcination to form carbon nanosheets with a strong network. The same process in the presence of pyridine and carbon nanorings produced *N*-doped carbon nanosheets with an unexpectedly high nitrogen content. X-ray photoelectron spectroscopy (XPS) showed that the nitrogen in these carbon nanosheets has an electronic state that exhibits useful catalytic activity. The thin film fabrication method used in this study requires only a very small amount of carbon nanorings (1 ng) to fabricate a 1 m^2^ nanosheet. Furthermore, the technology can be industrially deployed by increasing the area of the nanosheet to a large area. In particular, the synthesized *N*-doped carbon nanosheets are expected to be used for various applications, such as efficient catalysts for oxygen reduction reactions in high-performance fuel cells and high-performance electrochemical supercapacitors. In addition, their mesoporous structure with a large surface area is expected to be applied to fuel cells and other applications as a catalyst that can replace expensive platinum.

**Figure 11:**
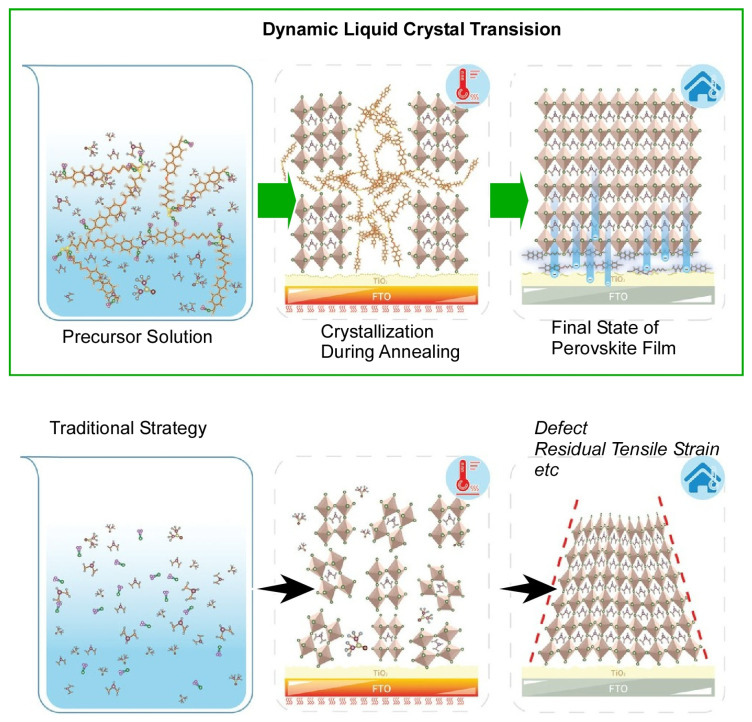
Dynamic liquid crystal transition using interfaces designed to spontaneously delay bulk crystallization and release residual strain at the interface in situ during annealing, spontaneously healing the interface via a dynamic transition to give better perovskite materials. Reprinted with permission from [393]. Copyright 2022, Wiley-VCH.

Low temperature solution processes for thin film semiconductors are more cost-effective than conventional vacuum processes. However, they lead to more defects and residual tensile stress during high-speed bulk crystallization. Chen, You, Liu, and co-workers have developed a new strategy called dynamic liquid crystal transition using interfaces (Figure 11) [393]. The above problem can be solved in one step. The new strategy, dynamic liquid crystal transition, is designed to spontaneously delay bulk crystallization and release residual strain at the interface in situ during annealing. In the design principle of perovskite thin films using this method, the dynamic liquid crystal transition molecules first interact with the bulk perovskite crystal grains, while spontaneously healing the interface via a dynamic transition. Thermotropic liquid crystal molecules are used to demonstrate this strategy. Liquid crystal molecules interacting with perovskite colloids form intermediate adducts that delay the crystallization. The interaction of lead iodide with the target molecules in the solution delays the crystallization process and increases the crystal grain size. During the dynamic transition of the target molecules, they leave the perovskite and attach to the carrier transport layer. Target molecules concentrated at the interface tune the thermal mismatch. They act as a buffer to release residual strain between the perovskite and the carrier transport layer. This technique also significantly improves the environmental stability and photostability of the perovskite. These techniques envision a new engineering method of additive phase transitions for high-performance perovskite solar cells. The dynamic liquid crystal transition strategy can be a general method to achieve both delayed crystallization and spontaneous lower interface healing in a single step in the solution processing of thin film semiconductors.

**Figure 12:**
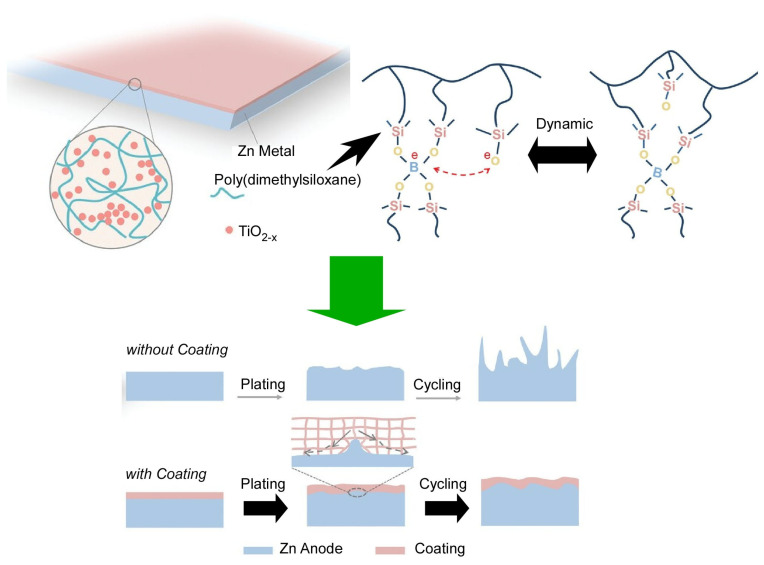
Self-adaptive polydimethylsiloxane/TiO_2−x_ coating that can dynamically adapt to volume changes and inhibit dendrite growth with high dynamic adaptability due to the micro-crosslinking of the B-O bonds. Reprinted with permission from [406]. Copyright 2022, Wiley-VCH.

Various batteries, such as lithium-ion batteries [394,395,396,397,398] and potassium-ion batteries [399,400,401], have attracted significant attention. Zinc-ion batteries have the advantage of high safety and have also attracted attention from those focused on environmental protection [402,403,404,405]. However, there is the critical problem of dendrite formation and the rapid decrease in battery life due to Zn debris that accumulates on the surface. To resolve this problem, Fan, Zhang, and co-workers developed a self-adaptive polydimethylsiloxane/TiO_2-x_ coating that can dynamically adapt to volume changes and inhibit dendrite growth (Figure 12) [406]. The polymer synthesized here, polydimethylsiloxane, has high dynamic adaptability due to the micro-crosslinking of B-O bonds. There is sufficient time for the B-O bonds to break, and entanglement of the molecular chains prevents molecular deformation. Therefore, polydimethylsiloxane is fluid on the macroscale. With an increasing strain rate, the characteristic breaking time of the B-O crosslink becomes dominant. The B-O bonds resist the movement of the molecular chains and exhibit some degree of elasticity. The combination of TiO_2−x_ with high oxygen vacancies and polydimethylsiloxane induces rapid and uniform migration of Zn^2+^. The coated anode is characterized by excellent cycling stability and shows great potential as an anode for Zn ion batteries. This nanoarchitectonics of dynamically adapting artificial coatings may be a beneficial method for the protection of various electrodes.

As seen in molecular manipulation and molecular assembly, anisotropic assemblies of materials are formed at dynamic interfaces. Under such conditions, reactions such as covalent bonding can produce ultrathin film forms with unique structures such as ordered porous materials including metal–organic frameworks (MOFs), covalent organic frameworks (COFs), and nanocarbon ultrathin films. Dynamic interfaces are an ideal place for the production of nanocarbon ultrathin films. At dynamic interfaces, reactants from both phases that form the interface dynamically come together to form the material. There is tremendous potential for the selection of components. The products often remain at the interface and can be easily recovered. Many possibilities remain in this area, and it is expected to develop not only in academic research but also in industrial applications.

## 4. Fullerene Assembly: Examples of Dynamic Formation Process from Zero-Dimensional Unit to Advanced Materials

An interesting example of nanoarchitectonics at dynamic interfaces is the formation of supramolecular assemblies at the liquid–liquid interface of fullerenes (C_60_, C_70_, etc.). When a fullerene is dissolved in a well-soluble solvent and a fullerene-poor solvent is added, a variety of supramolecular assemblies and crystals are formed at the liquid–liquid interface [407]. Fullerenes are composed of a single element, carbon, and have a simple zero-dimensional shape. Nevertheless, by simply changing the conditions, one-dimensional [408,409], two-dimensional [410,411], three-dimensional [412,413], and hierarchical structures [414] can be obtained. Those structures can also be treated at high temperatures to synthesize various forms of carbon materials. These systems reflect the diversity of nanoarchitectonics at dynamic interfaces. In the following, some recent examples are given.

**Figure 13:**
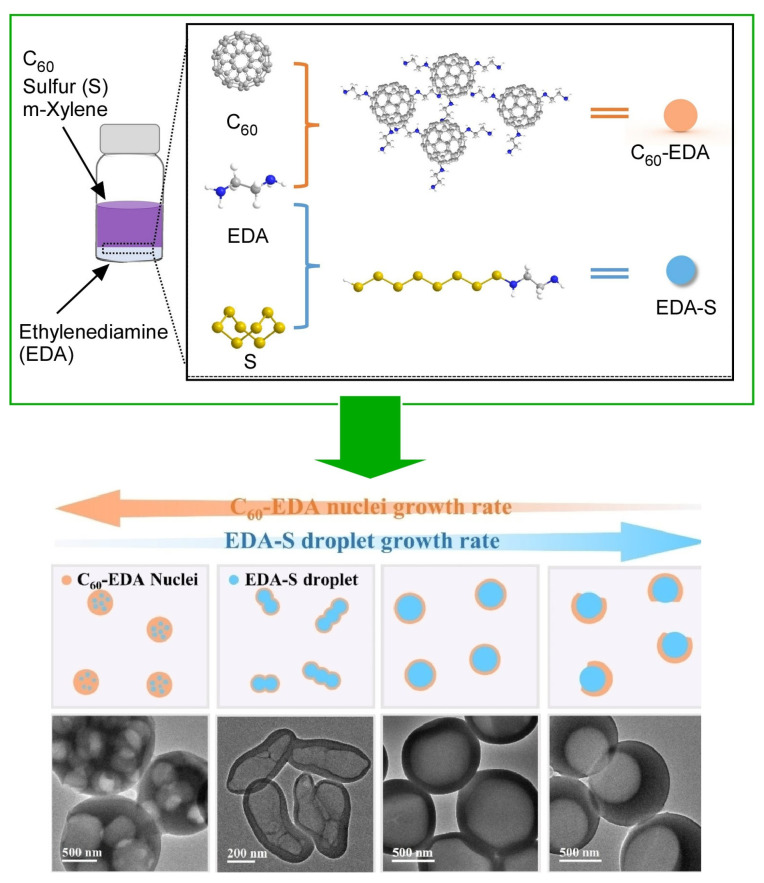
The kinetically controlled liquid–liquid interfacial precipitation (KC-LLIP) strategy, where ethylenediamine was selected as a covalent cross-linker of C_60_ and the formation of C_60_-ethylenediamine products was controlled by the addition of isopropyl alcohol. To introduce the hollow structure, ethylenediamine-sulfur was used as an in situ generated droplet to form the yoke–shell structure, giving porous spheres, string hollow spheres, hollow spheres, and aperture hollow spheres. Reprinted with permission from [418]. Copyright 2022, Wiley-VCH.

Hollow carbon spheres are widely expected to be used in a variety of technological fields, including energy conversion and storage, catalysis, adsorption, drug delivery, and nanodevices [415,416,417]. However, it is not always easy to control the properties and morphology of the resulting hollow carbon materials. Chen et al. have developed kinetically controlled liquid–liquid interfacial precipitation, in which solid or hollow C_60_ nanospheres are kinetically controlled liquid–liquid interfacial precipitation that can control morphology and size [418]. This strategy is called the kinetically controlled liquid–liquid interfacial precipitation (KC-LLIP) strategy (Figure 13), where ethylenediamine was selected as a covalent cross-linker of C_60_, and the formation of C_60_-ethylenediamine products was controlled by the addition of isopropyl alcohol. To introduce the hollow structure, ethylenediamine-sulfur was used as an in situ generated droplet to form the yoke–shell structure. The design of the hollow C_60_ spherical structures was based on controlling the growth rate of the ethylenediamine-sulfur yoke and the C_60_-ethylenediamine shell. Thus, porous spheres, string hollow spheres, hollow spheres, and aperture hollow spheres were obtained. Some examples are as follows. Immediate deposition of C_60_-ethylenediamine on the surface of the first dispersed ethylenediamine-sulfur droplet yields porous spheres. The ethylenediamine-sulfur droplet is successfully incorporated as a template, so that the interior of the porous sphere contains many pores. The formation of string-like hollow spheres occurs by adding an appropriate amount of isopropyl alcohol to the ethylenediamine solution to reduce the growth rate of C_60_-ethylenediamine and promote the diffusion of ethylenediamine-sulfur droplets in the *m*-xylene solution. Hollow spheres were obtained when ethylenediamine-sulfur droplets coalesced into larger spheres during C_60_-ethylenediamine aggregation. Excess isopropyl alcohol causes the rapid formation of deformed ethylenediamine-sulfur droplets, resulting in the formation of sealed deformed spheres. The formation of open-aperture hollow spheres is due to the inability of the C_60_-ethylenediamine nucleus to precipitate into the shell aperture if it is occupied by other ethylenediamine-sulfur droplets during the coalescence. These diverse nanoarchitectonics represent an effective strategy for synthesizing C_60_ spheres, whose dimensions and morphology can be tuned through various kinetic controls using droplets as templates. Moreover, this nanoarchitectonics approach is characterized by its ease of implementation on a large scale. The resulting C_60_ nanospheres can be applied as effective carbon materials for advanced applications.

**Figure 14:**
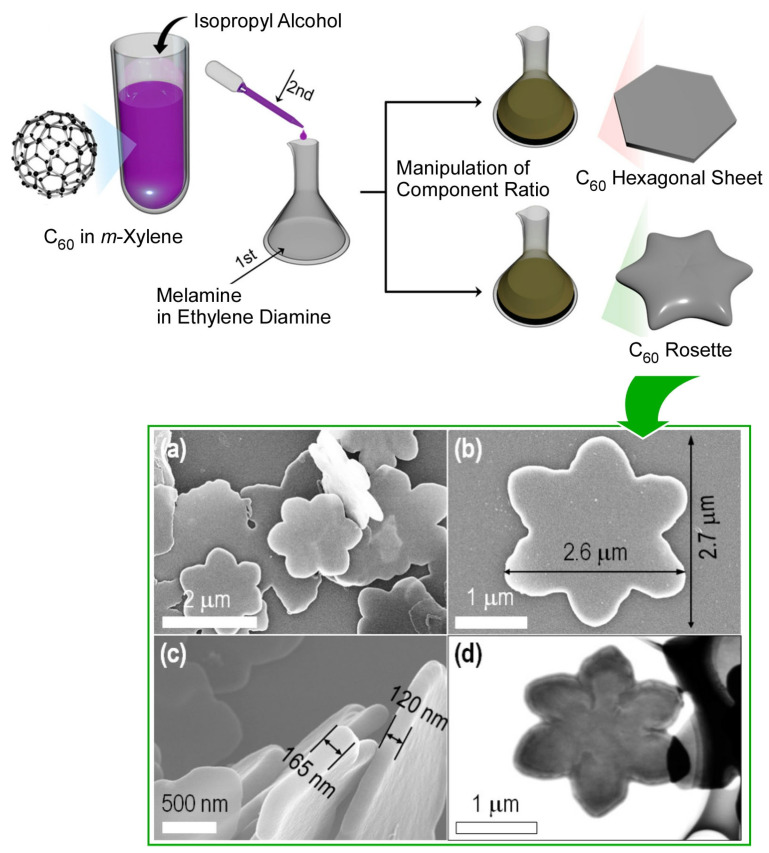
The in situ reaction method to the self-assembly process of C_60_ molecules and mela-mine/ethylenediamine components in solution, producing a new fullerene assembly, a micron-sized, two-dimensional, amorphous-shaped, regular object, a fullerene rosette (**a**–**d**). Reproduced under terms of the CC-BY license [420]. Copyright 2022, MDPI.

Certain amine reagents, such as ethylenediamine, are active reactants of fullerenes. Based on this activity, there is a technique for nanoarchitectonics of aggregates while reacting in situ. This technique has a high potential for the preparation of highly integrated structures and nitrogen-doped materials [419]. Chen et al. applied the in situ reaction method to the self-assembly process of C_60_ molecules and melamine/ethylenediamine components in solution (Figure 14) [420]. As a result, they succeeded in producing a new fullerene assembly, a micron-sized, two-dimensional, amorphous-shaped, regular object, a fullerene rosette. The two-dimensional fullerene rosette consists of six petals partially resembling hexagonal sheets, with a very smooth surface with thicknesses in the 120–165 nm range. No clear crystalline features in the two-dimensional plane were detected from the XRD patterns. The two-dimensional fullerene rosettes were immobilized in a quartz crystal microbalance to create sensors for a variety of volatile organic compounds. The frequency shifted quickly when exposed to volatile organic compounds. When the solvent vapor was removed from the chamber, it returned to almost its initial state. Thus, reversible vapor adsorption/desorption was suggested. The detection sensitivity for a wide range of volatile organic compounds obeys as follows, in order: formic acid > acetic acid > pyridine > acetone > aniline > benzene > ethanol > ethyl acetate > toluene > cyclohexane > hexane. Formic acid sensing was compared to other carbon materials, hexagonal nanosheets, fullerene nanotubes, acid-treated fullerene nanotubes, and commercial activated carbon. The two-dimensional fullerene rosette-modified quartz resonator electrode showed a remarkable performance compared to the other materials tested. The high ability to sense acid vapors such as formic and acetic acids is probably due to the abundance of amino groups in the aggregates. The rosette-based quartz crystal resonator sensor was also superior in the subtle size discrimination of formic and acetic acids. Another advantage of the developed fullerene rosette-based sensor is that it can be fabricated and measured at room temperature, avoiding a high temperature processing.

**Figure 15:**
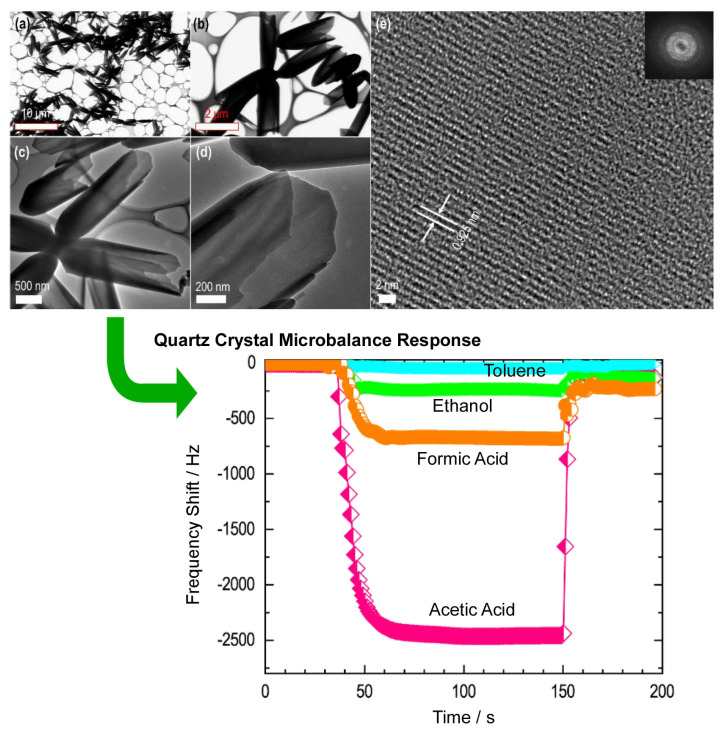
Cone–husk fullerene C_60_ crystals prepared by dynamic liquid–liquid interface precipitation under ambient temperature (**a**–**e**) and pressure with high sensitivity to acetic acid in quartz crystal resonator sensor electrodes modified with cone–husk fullerene C_60_ crystals (bottom). Reproduced under terms of the CC-BY license [421]. Copyright 2022, MDPI.

Wei at al. reported the vapor sensing performance of self-assembled, cone–husk fullerene C_60_ crystals prepared by dynamic liquid–liquid interface precipitation under an ambient temperature and pressure (Figure 15) [421]. Due to the unique semi-open tubular structure and typical micropore size, the cone–husk fullerene C_60_ crystals exhibit a highly sensitive detection performance for acetic acid vapor. The object uses a very quick liquid–liquid interfacial precipitation method. Isopropyl alcohol was quickly added to a fresh saturated solution of C_60_ in mesitylene and immediately shaken vigorously by hand for about 3 s. The cone–husk fullerene C_60_ crystal precipitate was washed with isopropyl alcohol to remove the organic solvent. They were separated from the mixture by centrifugation and dried in a vacuum oven at 70 °C for 3 h. Electron microscopic analysis confirmed that the cone–husk fullerene C_60_ crystals have a unique thin tubular wall with a semi-open structure at one end and a solid structure at the other end. Cone–husk fullerene C_60_ crystals have a microporous structure with crystalline pore walls. This structure provides the nanospace necessary for the adsorption of guest vapors. As a result, the gas-sensing performance is enhanced. The sensitivity of quartz crystal resonator sensor electrodes modified with cone–husk fullerene C_60_ crystals is in the following order: acetic acid > formic acid > ethanol > water > acetone > pyridine > formaldehyde > methanol > toluene > aniline > cyclohexane > hexane > 2-propanol. The diffusion of acetic acid vapor is facilitated by the unique thin tubular wall with a semi-open structure at one end of the cone–husk fullerene C_60_ crystal, which facilitates the diffusion of acetic acid vapor. This provides the nanospace necessary for the adsorption of acetic acid guest vapor and improves the gas-sensing performance. Cone–husk fullerene C_60_ crystals have great potential in the development of systems for volatile organic compound sensing that are selective for aliphatic acid vapors.

**Figure 16:**
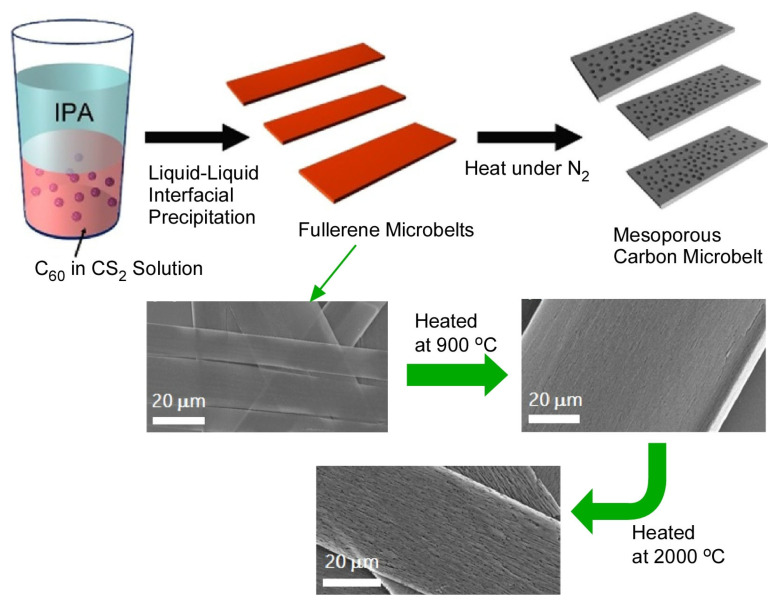
Supramolecular assembly of fullerene C_60_ into high aspect ratio quasi-two-dimensional microbelts at room temperature at the dynamic liquid–liquid interface of a carbon disulfide solution of fullerene C_60_ and isopropyl alcohol. The formed fullerene microbelt can be converted to a mesoporous carbon microbelt with amorphous or graphite backbone structure through carbonizing at 900 °C or 2000 °C, respectively. Reprinted with permission from [422]. Copyright 2017, American Chemical Society.

Tang et al. achieved a supramolecular assembly of fullerene C_60_ into high aspect ratio quasi-two-dimensional microbelts at room temperature at the dynamic liquid–liquid interface of a carbon disulfide solution of fullerene C_60_ and isopropyl alcohol (Figure 16) [422]. The fullerene microbelt was synthesized using the static liquid–liquid interfacial precipitation method. The length of the fullerene C_60_ microbelt can be controlled by the synthesis conditions. By optimizing the conditions, a fullerene microbelt with a length of 1 cm could be synthesized. The quasi-two-dimensional-structured fullerene microbelt was expected to have excellent mechanical stability, flexibility, transparency, and chemical stability. The fullerene microbelt can be converted to a mesoporous carbon microbelt with an amorphous or graphite backbone structure through carbonizing at 900 °C or 2000 °C, respectively. Nanoarchitectonics by high temperature heat treatment results in a novel bimodal porous carbon material derived from fullerene crystals, a crystalline p-electron rich carbon source. It exhibits higher specific capacitance than graphene, conventional mesoporous carbon, and other similar carbon materials, and it demonstrates an excellent electrochemical supercapacitor performance. The quasi-two-dimensional microbelt morphology with large pore volume is advantageous for enhanced charge transport in supercapacitor electrodes. They can be a potentially excellent material for energy storage applications.

Self-assembly and subsequent material creation at dynamic interfaces using fullerenes have been reported far beyond what is shown here. It is astonishing that fullerene, a single element, zero-dimensional object, a nanounit without anisotropy or specific functional groups, can create such a wide variety of materials. It shows the great potential of nanoarchitectonics, especially dynamic interfacial nanoarchitectonics.

## 5. Summary and Perspectives

In this review, nanoarchitectonics at dynamic interfaces as a method to create more functional materials has been discussed. Nanoarchitectonics is a methodology to architect functional materials using nanounits such as atoms, molecules, and nanomaterials. Since the method is applied without limiting the materials, it can be generally applied to almost all materials. A similar architecture and organization of functional structures is often seen in biological systems. In these systems, the interfacial environment and dynamic properties are strongly expressed. As a result of this background, therefore, it is suggested that nanoarchitectonics at dynamic interfaces is a promising method for the development of functional materials.

To this end, liquid interfaces provide a beneficial environment. Compared to crystals and solid surfaces, liquid interfaces have a much higher degree of freedom of motion for the molecules and materials contained therein. Furthermore, since the range of motion and dispersion is limited to a two-dimensional system, it is more controllable than a solution system spread over three dimensions. The general trends of the corresponding developments can be summarized with the listed examples. Liquid interfaces, such as gas–liquid and liquid–liquid interfaces, are important fields for nanoarchitectonics because these interfaces have a high degree of freedom in terms of their responsiveness to external stimuli. Their dynamic functions can also be controlled. Molecular manipulation and the formation of specific molecular aggregates are performed by external forces. In the example discussed above, luminescence behavior can be controlled through manipulating the molecular position between the two phases at the air–water interface. Alternatively, the direction and intensity of the vortex flow at the interface can tune the circularly polarized luminescence of the assembly style of the functional complex molecules. When such assembled molecules are reacted or combined, they become functional materials. In other words, the dynamic interface is a suitable field for material production. For example, it is an ideal site for the fabrication of metal–organic frameworks (MOF), covalent organic frameworks (COF), and nanocarbon ultrathin films. At dynamic interfaces, the reactants from both phases that form the interface dynamically come together to form materials. There is tremendous potential for the selection of components. Furthermore, it has been demonstrated that a wide variety of structures and materials can be created by the nanoarchitectonics of fullerenes, which are units composed of a single element, carbon, and having a simple zero-dimensional shape. As a method itself, the nanoarchitectonics of dynamic interfaces has the power to create a diverse group of materials. In addition to that, there is a high degree of freedom in the selection of components at the interface, with the result that it has tremendous potential. In addition to academic research, the development of industrial applications is also expected.

The nanoarchitectonics of dynamic interfaces has many possibilities for development. One of them is its application to artificial functional structures such as devices. In such fields, top–down processing technologies such as microfabrication have been applied [423,424,425]. This can be converted to a bottom–up approach through introducing nanoarchitectonics at dynamic interfaces. In this case, many functional molecules and their supramolecular structures will be used for device fabrication [426,427,428,429,430]. Recently, Ishii, Yamashita, and co-workers have developed a chemical doping technique based on the redox reaction of benzoquinone and hydroquinone in an aqueous solution under ambient conditions [431]. The doping levels varied with the pH of the aqueous solution, and the conductivity was accurately and consistently controlled over a wide range of about five orders of magnitude. This was performed at polymer organic semiconductor thin film interfaces but could also be applied to dynamic liquid interfaces. When such semiconductor control techniques are coupled with the concept of nanoarchitectonics at dynamic interfaces, bottom–up device production becomes a reality. The history of the device industry, which has relied on physical fabrication techniques, can be rewritten due to nanoarchitectonics.

Another possibility is the challenge of building material systems in which many types of functional molecules are rationally organized, as is the case in biological systems. Until now, supramolecular chemistry has produced an organization of a relatively small number of types of molecules. By combining them, it will be possible to build complex and hierarchical functional architectures. However, this is not an easy task. Each set of components requires its own conditions and molecular design. It is not easy to optimize a large set of such components. Fortunately, humanity has developed artificial intelligence and incorporated it into materials science. For example, machine learning has proven to be one of the solutions to the search for answers in materials and chemistry research [432,433,434,435,436]. The related concept of materials informatics has also been proposed [437,438,439]. In fact, there are proposals to integrate nanoarchitectonics and materials informatics in the development of functional porous materials and others [440,441]. The ultimate goal of nanoarchitectonics is to create complex and highly functional material systems such as those found in biological systems. As discussed in this review, the interfacial environment and dynamic behavior are very important factors. In addition, the incorporation of new methods such as materials informatics is essential for the further development of nanoarchitectonics in the future. One of the remaining challenges is the application of these strategies at larger industrial scales. Therefore, technological advancements must also be considered as important consequences.

## Data Availability

Data sharing not applicable.
